# Mechanisms for regulation of RAS palmitoylation and plasma membrane trafficking in hematopoietic malignancies

**DOI:** 10.1172/JCI171104

**Published:** 2023-06-15

**Authors:** Fang Yu, Zhijian Qian

**Affiliations:** 1 Department of Medicine, UF Health Cancer Center, and; 2 Department of Biochemistry and Molecular Biology, University of Florida, Gainesville, Florida, USA.

## Abstract

Palmitoylation is a critical posttranslational modification that enables the cellular membrane localization and subsequent activation of RAS proteins, including HRAS, KRAS, and NRAS. However, the molecular mechanism that regulates RAS palmitoylation in malignant diseases remains unclear. In this issue of the *JCI*, Ren, Xing, and authors shed light on this topic and revealed how upregulation of RAB27B, as a consequence of CBL loss and Janus kinase 2 (JAK2) activation, contributes to leukemogenesis. The authors found that RAB27B mediated NRAS palmitoylation and plasma membrane localization by recruiting ZDHHC9. The findings suggest that targeting RAB27B could provide a promising therapeutic strategy for NRAS-driven cancers.

## RAS trafficking and activation by posttranslational modifications

Three human RAS genes encode four highly homologous proteins: NRAS, KRAS4a, KRAS4b, and HRAS, which share about 85% identity and a conserved catalytic domain (1). Oncogenic RAS mutations are found in approximately 25% of human cancers and are also prevalent in myeloid disorders (2, 3). The most common dominant somatic mutations in RAS genes occur at codons 12, 13, and 61, and substitution at any of these residues induces RAS proteins as constitutively active in human cancers by favoring the GTP-bound conformation. Different RAS genes are preferentially mutated in distinct cancer types, with KRAS mutations highly prevalent in common epithelial malignances (4). Hematologic cancers are unusual in that NRAS and KRAS are both mutated at high frequencies, with NRAS mutations predominating (5). Numerous studies suggest that RAS mutations are prevalent in relapsed hematological malignancies and that they are associated with poor overall survival. However, the direct targeting of RAS proteins remains a substantial challenge due to a lack of druggable pockets on the protein surface and a picomolar affinity for binding with GTP. Consequently, there is a need to find alternative strategies that disrupt RAS signaling.

RAS family proteins share the amino acid sequence CAAX in their carboxyl-terminal, where C is cysteine, A is usually aliphatic, and X is any amino acid (6). The CAAX motif always carries a prenyl or farnesyl posttranslational moiety, termed CAAX processing, which is necessary, but not sufficient, for delivery of RAS proteins to the plasma membrane. RAS family proteins also must undergo a series of posttranslation modifications at their C-terminal end for differential targeting to distinct membranes and activation, called a second signal, and occurs upstream of the CAAX motif (7). Palmitoylation of RAS, as one of the second signals, can confer RAS proteins with a 100-fold greater affinity for membranes than prenylated-only proteins (8, 9). Protein palmitoylation is dynamically controlled by palmitoyl-acyl transferases and palmitoyl thioesterases. Palmitoylation regulates protein folding in the ER, mediates protein retention in the Golgi, and determines protein interaction with specific membranes or membrane domains (10).

Like all RAS proteins, NRAS undergoes a series of posttranslational modifications necessary for binding to the plasma membrane and subsequent activation of downstream signaling pathways. The requirement for palmitoylation of RAS proteins in NRAS-driven leukemia has been demonstrated in the NRAS^G12D^ model, which reflects a common NRAS mutation in human myeloid malignancies (11, 12). However, another study that used a KRAS^G12D^ model observed that palmitoylation was not essential in KRAS-mediated leukemogenesis (13).

In this issue of the *JCI*, Ren, Xing, et al. (14) provided compelling evidence that palmitoylation of NRAS plays a critical role in trafficking NRAS to the plasma membrane and activating NRAS signaling. The findings suggest that targeting RAB27B by blocking the palmitoylation of NRAS could provide a promising therapeutic strategy for NRAS-driven cancers.

## CBL/JAK2/RAB27B signaling axis

Three members of CBL family ubiquitin E3 ligases have been identified so far, namely C-CBL (also called CBL)**,** CBL-B and CBL-C. Previous studies have shown that deletions or loss-of-function mutations in CBL are frequently observed in myeloid malignancies, especially in myelodysplastic syndrome/myeloproliferative neoplasm (MDS/MPN) overlap syndromes (15, 16). Janus kinase 2 (JAK2) is a pivotal kinase in hematopoietic stem and progenitor cells (HSPCs), and its uncontrolled hyperactivation is a prominent oncogenic driver of hematopoietic neoplasms (17). Nonetheless, the mechanisms behind CBL loss and JAK2 activation–mediated leukemogenesis are not fully understood. Previous research from Dr. Wei Tong’s laboratory has shown that ubiquitination of JAK2 by CBL and CBL-B regulates the stability and activity of the JAK2 protein, playing a critical role in curbing HSPC expansion and preventing the development of myeloid malignancies (18). Ren, Xing, et al. (14) present further evidence supporting their previous findings. They demonstrated that CBL loss resulted in the stabilization of JAK2 protein, which, in turn, promoted the transcription of RAB27B. They also found that RAB27B was upregulated in patients who had a myeloid malignancy that carried CBL or JAK2 mutations; and RAB27B levels correlated with a poor prognosis.

Ren, Xing, et al. provide further insights into the mechanism of palmitoylation of NRAS, which was mediated by the CBL/JAK2/RAB27B signaling axis (14). The findings also suggested that CBL loss activated both NRAS and KRAS, whereas knockdown of RAB27B only prevented CBL loss–mediated activation of NRAS but not of KRAS. Finally, the Ren, Xing, et al. (14) study showed that knockdown of CBL dramatically enhanced palmitoylation of NRAS but not of KRAS, indicating that additional mechanisms responsible for CBL/NRAS mutation-driven hematopoietic malignancies likely exist. Since palmitoylation of RAS is regulated and revisable, RAS palmitoylation offers another layer for therapeutic intervention. A genome-wide CRISPR/Cas9 screening for proteins that regulate the palmitoylation of RAS would further expand this treatment potential and provide a valuable avenue for future studies.

## RAB27B regulates NRAS signaling by controlling NRAS palmitoylation

The small RAB GTPases RAB27A and RAB27B have overlapping and unique roles in regulating intracellular vesicle trafficking, docking, and fusion with the plasma membrane. It is noteworthy that only RAB27B has been found to be highly expressed in solid cancers. This high expression of RAB27B correlates with metastasis and poor survival (19, 20). Additionally, RAB27B overexpression is associated with a poor prognosis in AML patients. However, the precise role of RAB27B in hematopoietic malignancies remains unclear. Ren, Xing, et al. (14) reveal a role for RAB27B in hematopoietic malignancies, particularly in NRAS mutant-driven myeloid malignancies. The findings suggest that RAB27B plays a critical role in promoting oncogenic NRAS signaling and leukemic growth, and inhibiting RAB27B may represent a potential therapeutic strategy. The study also highlights the importance of identifying small molecules that can disrupt the interaction between RAB27B and ZDHHC9 as a potential treatment approach for NRAS mutation–driven hematopoietic malignancies (14).

## Conclusion

**Figure 1:**
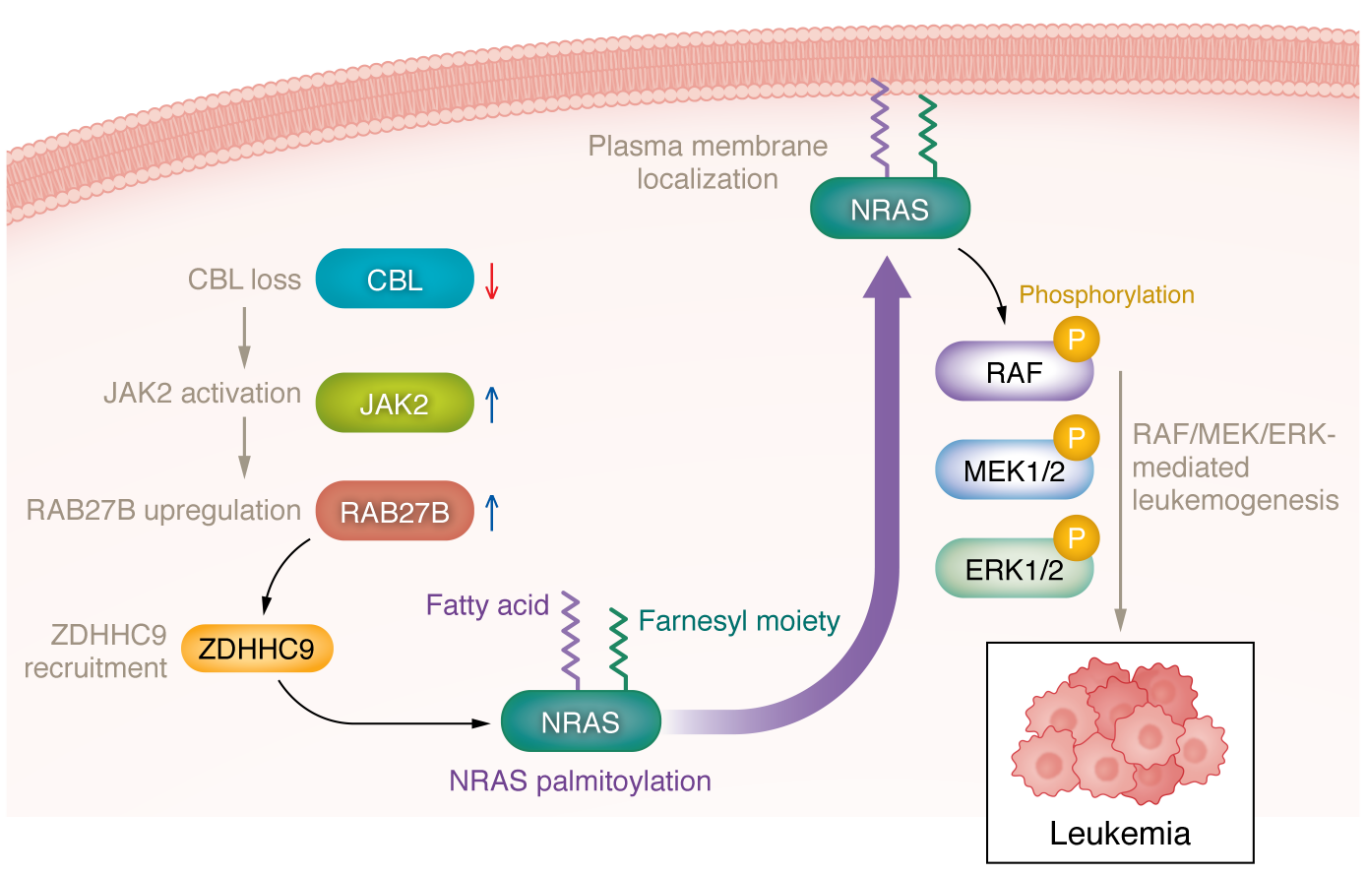
The proposed mechanism for the regulation of NRAS mutation-driven hematopoietic malignancies involves the CBL/JAK2/RAB27B signaling axis. Deletion or loss-of-function mutations of *CBL* stabilize JAK2, leading to upregulation of the transcription of *RAB27B*. RAB27B facilitates NRAS trafficking to the plasma membrane and regulates its palmitoylation, thereby activating the RAS/RAF/MEK/ERK signaling pathway and promoting leukemogenesis.

Ren and colleagues have discovered a role of the CBL/JAK2/RAB27B/ZDHHC9 signaling axis in regulating NRAS trafficking to the plasma membrane for activation by palmitoylation in myeloid malignancies (14) (Figure 1). This study presents the possibility for targeting RAS-driven cancers by specifically blocking NRAS palmitoylation. However, it also raises important unknowns. For instance, the specific palmitoylation sites of NRAS and the palmitoyl thioesterases of NRAS have yet to be identified in leukemia. Furthermore, the prevalence of simultaneous CBL loss and NRAS mutations in hematopoietic malignancies is unclear. Finally, the precise mechanism by which RAB27B facilitates the palmitoylation of NRAS still requires additional investigation. Further exploration will reveal the detailed mechanisms underlying the role of RAB27B in RAS-driven cancers and the therapeutic potential of targeting RAB27B in the treatment of hematopoietic malignancies.

## Version 1 06/15/2023

Electronic publication
